# Measuring the efficiency of public hospitals: A multistage data envelopment analysis in Fujian Province, China

**DOI:** 10.3389/fpubh.2023.1091811

**Published:** 2023-03-07

**Authors:** Mengya Sun, Yaojun Ye, Guangdi Zhang, Yuan Xue, Xiuling Shang

**Affiliations:** ^1^College of Science, Zhejiang University of Science and Technology, Hangzhou, China; ^2^Operation and Management Office, Fujian Provincial Hospital, Fuzhou, China; ^3^The Third Department of Critical Care Medicine, Shengli Clinical Medical College of Fujian Medical University, Fujian Provincial Hospital, Fujian Provincial Center for Critical Care Medicine, Fujian Provincial Key Laboratory of Critical Care Medicine, Fuzhou, China

**Keywords:** hospital efficiency, gray relational analysis, gray clustering, data envelopment analysis, Tobit regression

## Abstract

**Objective:**

The present study aimed to evaluate the operational efficiency of public hospitals in Fujian Province and the factors responsible for the inefficiency of these hospitals and provide relevant suggestions for health policymakers in allocating service resources.

**Method:**

In the first stage of the research, the variables affecting the efficiency of hospitals were extracted by qualitative and quantitative methods, including literature optimization, gray related analysis and gray clustering evaluation. In the second stage, the data envelopment analysis (DEA) method was used to evaluate the operational efficiency of 49 hospitals of different levels and types selected by sampling in 2020. Finally, a Tobit regression model with introduced institutional factors and background factors was established to study the main influencing factors of hospital inefficiency.

**Results:**

In the first stage, 10 input variables and 10 output variables necessary from the mangers' point of view were identified to test efficiency. In the second stage, the average comprehensive TE, PTE, and SE of 49 sample hospitals was 0.802, 0.888, and 0.902, respectively. 22.45% of these hospitals met the effective criteria, i.e., the overall effective rate was 22.45%. The low SE value of the hospital was the main reason hindering the improvement of the comprehensive efficiency value. The overall effective rate of secondary public hospitals (30.77%) was higher than that of tertiary public hospitals (19.44%), and the overall effective rate of public specialized hospitals (30%) was higher than that of general public hospitals (18.92%). Based on the third stage results, the bed occupancy rate (BOR) and the proportion of beds (POB) were major factors affecting the operation efficiency of grade III hospitals (*p* < 0.01). However, the operating efficiency of grade II hospitals was significantly affected by POB and regional per capita GDP(GDPPC) (*p* < 0.05). Moreover, the impact of BOR and GDPPC was positive, and POB was negatively correlated with hospital operation efficiency.

**Conclusions:**

The study results indicated that the overall operation efficiency of public hospitals in Fujian Province is low. This study revealed that intervention should be strengthened from a policy and management perspective to improve the operation efficiency of public hospitals.

## 1. Introduction

Since the launch of medical reform, the Chinese government has increased its investment in medical and health services and implemented a series of effective reform measures. However, the issue of “high expense and difficulties in medical care” is persistent. In 2013, Hu et al. (1) reported inefficiencies in the allocation of health resources and service delivery in China. The contradiction caused by the uneven distribution of medical resources has become a potential threat to social stability.

As the main body of China's medical service system, the reform of public hospitals is a major part of the reform of China's medical and health system. The development of public hospitals plays a critical role in continuously improving the fairness and accessibility of basic medical and health services, preventing and controlling major epidemics such as COVID-19, and ensuring the safety and health of the public (2). In 2021, China's policy to promote the high-quality development of public hospitals improved hospital efficiency and saved costs to reduce the burden of patients seeking medical treatment. The efficiency of public hospitals directly affects the level of medical supply.

In order to further rationally allocate and utilize medical resources and improve medical efficiency, Fujian Province issued the Public Hospital Quality Information Disclosure Plan, which requires the secondary and tertiary public hospitals to disclose medical resource allocation, medical expenses and other indicators to the public quarterly to increase the transparency of medical services and promote further improvement of medical services (3). This plan provides a reference for other cities to promote hospital reform. Public hospitals in China are divided into three levels according to the number of beds. Grade I hospitals have beds <100, secondary hospitals have beds between 100 and 500, and tertiary hospitals have beds >500 (4). The service scope of different levels of hospitals is different. Comparing the operation efficiency of public hospitals at different levels can promote the improvement and perfection of public hospitals and provide basis for promoting the high-quality development of public hospitals.

Globally, the measurement of hospital efficiency has been achieved through various technologies. Aigner and Chu (5) were among the first researchers to estimate the production frontier using ordinary least squares analysis. However, this model was criticized soon because the entire distance between the production frontier and each individual observation was attributed to low efficiency. Initially, Cobb Douglas production function was widely used because it was simple to analyze and could interpret estimated input coefficients as partial elasticity (6). However, it has been criticized for the imposition of the elasticity of input substitution equal to 1 and the forced implementation of fixed-scale economy (7). A more flexible specification is the Translog production function, which allows variable scale efficiency and variable elasticity of substitution. However, the introduction of a large number of additional parameters may lead to a large loss of multicollinearity and degrees of freedom (8). Aigner et al. (9) proposed a stochastic frontier model, which adds an additional random variable Vi to the inefficiency variable ui. This takes into account the influence of measurement errors and other stochastic elements. The disadvantage is that it depends on strong distribution assumptions to distinguish whether the residual error in the regression is caused by noise or technical efficiency. Charnes et al. (10) proposed a non-parametric method DEAwhich measures efficiency with efficiency frontier, and obtains efficiency frontier with the help of linear programming model (11). The biggest advantage of DEA method is that it does not need to specify the production function. In addition, it can consider multiple inputs and outputs at the same time (12). Currently, DEA is the most analytical method for evaluating the medical performance in healthcare-related fields (13).

Recent studies have measured and studied the medical efficiency in practical applications from different dimensions. First, research from the national dimension focuses on measuring the efficiency of different countries (14, 15). For example, Aydin, A. identified the efficiency of health care services in OECD countries (16, 17). Top, M. et al. measured the healthcare system efficiency of 36 African countries (18). Second, some scholars study the efficiency in different regions of one country (19–21). For example, Mazon assessed the technical efficiency of municipalities of the State of Santa Catarina in public health expenditures and its relationship with health management conditions (22). Ngobeni assessed and compared the technical efficiency of the nine South African provinces in the provision of healthcare (23). In addition, some scholars study the efficiency of hospitals with different characteristics (24, 25), such as teaching and non-teaching hospitals in the United States (26), general hospitals, specialized hospitals, and Multi-specialized hospitals in southwest of Iran (27). In the case of China, Gong et al. evaluated the overall and two substage efficiencies of China's healthcare system in each of its province (28). Du analyzed the association between quality and efficiency from each group of the national, east, central and west (29). Jing et al. (30) evaluated the technical efficiency of public and private hospitals in Beijing, China. After the reform of China's medical system, the public sector have been concerned about the efficiency of primary health services (31–34), while ignoring the services of hospitals above the second level.

In the data envelopment analysis method, the quality of indicators selected has a serious impact on the research results. In the current research, most of the indicators are selected using qualitative methods (35, 36), such as the Delphi method (37). Some scholars use quantitative methods to select indicators, such as principal component analysis (PCA) (38) and efficiency contribution measurement (ECM) (39). Qualitative methods are highly subjective and need to be used in combination with quantitative methods to ensure the objectivity of indicators.

In the research of medical system, most of them use traditional or improved DEA, such as Lari and Sefiddashti (40). Some scholars combine DEA model with other methods, such as Malmquist index (41). Most of these methods are used to measure the change of efficiency value. This paper focuses on exploring the mechanism of hospital operating efficiency, finding the determinants of low efficiency value, and providing a basis for improving hospital operating efficiency. Therefore, this paper combines Tobit regression model with traditional DEA model to find the influencing factors and differences of hospital efficiency at different levels.

Considering the current research, the purpose and innovation of this paper are as follows. First of all, the scientific efficiency evaluation index system is constructed by combining the gray correlation and gray clustering methods with the literature optimization method. Secondly, the two-stage DEA model is used to measure the operation efficiency of different levels of hospitals, and explore the influencing factors and differences of the efficiency of different levels of hospitals, so as to provide reference for deepening the quality development of public hospitals.

## 2. Materials and methods

In this study, a two-stage efficiency analysis was performed in cross-sectional data. In the first stage, DEA was used to estimate the efficiency scores of public hospitals in Fujian Province. In the second stage, Tobit regression analysis was used to identify the factors related to the efficiency of public hospitals. While the DEAP 2.1 program was used in the analysis of the efficiency scores of public hospitals, the Stata 16 program was used for the Tobit regression analysis.

### 2.1. Data source

This study selected public hospitals in Fujian Province as objects. Due to a large number of undisclosed samples of data, grade I hospitals were not included in this study. At the end of 2020, there were 229 public hospitals above level two in Fujian Province. Considering the requirements of data availability and sample size of the study, we selected 49 sample hospitals from 8 cities in Fujian Province by stratified sampling, of which the sample has the same structure as the population.

The indicator data were obtained from the statistical data of public hospital information disclosure indicators, the annual hospital department final account information published on the official websites of municipal governments, and health commissions in Fujian Province in 2020.

### 2.2. Methods

#### 2.2.1. Literature optimization method

First, the input and output indicators used frequently in previous studies were listed. Ozcan et al. (42) developed three categories of hospital input indicators, including capital investment, labor, and operating expenses. The output indicators were divided into two categories, including medical service operation and economic benefit. The database of alternative indicators was established based on the availability of data (Table 1).

**Table 1:** Database of hospital management efficiency indicators.

**Category of indicator**	**Evaluation indicators**
**Input indicators**	
Labor force indicators	*X*1: number of active staff; *X*2: number of physicians, *X*3: number of nurses, *X*4: ratio of doctors to nurses
Capital investment indicators	*X*5: number of hospital beds; *X*6: average number of open beds
Operating expense indicators	*X*7: total expenditure, *X*8: personnel expenditure, *X*9: utility expenditure, *X*10: project expenditure
**Output indicators**	
**Indicators of medical services**	
Resource allocation output	*Y*1: outpatient visit, *Y*2: discharge
Quality and efficiency	*Y*3: bed occupancy rate, *Y*4: number of bed turnover, *Y*5: average length of stay
**Economic efficiency indicators**	
Patient burden	*Y*6: average cost of outpatient (emergency) visits; *Y*7: average cost of hospitalization
Medical income	*Y*8: drug revenue, *Y*9: consumables revenue, *Y*10: inspection revenue and laboratory test revenue, *Y*11: medical service income
Capital income	*Y*12: total income, *Y*13: operating income, *Y*14: fiscal appropriation income

#### 2.2.2. Gray relational analysis and gray clustering evaluation

Since the operation of the health system was affected by various uncertainties, such as technical level and policy changes, it can be regarded as a gray system. Gray correlation analysis is an active branch of gray system theory that can compensate for the shortcomings caused by systematic analysis using mathematical-statistical methods. Gray correlation analysis does not require a specific size and regulation of the sample. Moreover, no inconsistency was detected between the quantitative and analysis results. Thus, gray correlation analysis can be used to select representative indicators.

In the database of alternative indicators, the evaluation indicators *Y*5, *Y*6, and *Y*7 were reverse indicators. The larger the indicators, the more detrimental they are to the efficient operation of the hospital, which was opposite to other indicators. Data envelopment analysis requires that the output and input indicators be coordinated. Thus, according to the needs of the model, the reciprocal method was applied to make *Y*5, *Y*6, and *Y*7 forward (Equation 1).


(1) $$\begin{array}{l} x_{ij}=\frac{1}{y_{ij}} \end{array}$$

wherein, *x*_*ij*_ is the forward indicator, *y*_*ij*_ is the reverse indicator.

Dyson et al. (43) emphasized that the number of input and output indicators should be streamlined, and the number of DMUs should be greater than twice the sum of the number of input and output indicators to ensure the effectiveness and stability of the model. Due to a large number of evaluation indicators, this study preliminarily screened the indicators by the coefficient of variation and the average value of the gray correlation degree of each evaluation index with other indicators of the same category. Supposedly, a category had m evaluation indicators and *n* evaluation objects, and the forward data matrix of the original data was expressed as (_*x*_*ij*_)*n*×*m*_. In order to compare the indicators of different dimensions, this study used the initialization operator to handle the data of each index using the dimensionless method (Equation 2).


(2) $$\begin{array}{l} z_{ij}=x_{ij}d=\frac{x_{ij}}{x_{i0}} \end{array}$$

in which *d* was the initialization operator. The normalized data matrix was expressed as (_*z*_*ij*_)*n*×*m*_.

One evaluation index was recorded as the reference series *Z*_0_. The gray relational degree between the remaining evaluation indicators and the reference series was calculated as follows (Equation 3); the above operation was repeated *n* times.


(3) $$\begin{array}{l} \gamma \left(z_{0j},z_{ij}\right)=\frac{\underset{i}{\min}\underset{j}{\min}|z_{0j}-z_{ij}|+\xi \;\underset{i}{\max}\underset{j}{\max}|z_{0j}-z_{ij}|}{\left|z_{0j}-z_{ij}|+\xi \;\underset{i}{\max}\underset{j}{\;}\max|z_{0j}-z_{ij}\right|} \end{array}$$

$$\begin{array}{l} \gamma \left(Z_{0},Z_{i}\right)=\frac{1}{n}\overset{n}{\underset{j=1}{\sum }}\gamma \left(z_{0j},z_{ij}\right) \end{array}$$

wherein ξ was the identification coefficient. The smaller the ξ, the higher the identification. ξ ∈ [0, 1], if ξ ≤ 0.5463, identification was the best at ξ = 0.5.

Finally, the coefficient of variation and the mean value of the gray correlation degree of each evaluation index with other indicators of the same category was calculated, wherein the mean value of the gray correlation degree was ε_*i*_ = γ(*Z*_0_, *Z*_*i*_)/(*m* − 1),*i* = 1, 2, ..., *m*. The larger the ε_*i*_, the more typical the indicator, the larger the coefficient of variation (CV), and the higher the sensitivity of the indicator.

In order to meet the principle of indicator refinement, this study used the gray clustering method to cluster the evaluation indicators and avoid selecting duplicate indicators. Finally, the gray relational degree, the coefficient of variation, and the clustering results were comprehensively considered to determine the selected evaluation indicators.

#### 2.2.3. Two-stage data envelopment analysis

Data envelopment analysis evaluated multiple inputs and outputs of the same type of decision making units (DMUs) simultaneously, and the operational efficiency of hospitals can be expressed as the weighted sum of hospital outputs to the weighted sum of hospital inputs (Equation 4).


(4) $$\begin{array}{l} Efficiency\;score=\frac{Weighted\;sum\;of\;hospital\;outputs}{Weighted\;sum\;of\;hospital\;inputs} \end{array}$$

The classical models widely used were mainly CCR and BBC. The CCR model proposed by Charnes et al. (10) assumes constant returns to scale (CRS) of production technology. However, the BCC model proposed by Banker et al. (44) speculated increasing returns to scale (IRS) of production technology to achieve technical efficiency without effects of size; it is also known as PTE.

The input and output data corresponding to *n* decision units were as follows:


$$\begin{array}{l} \begin{array}{c} x_{j}={\left(x_{1j},x_{2j},\cdots \;,x_{mj}\right)}^{T},\;j=1,2,\cdots \;,n,\\ y_{j}={\left(y_{1j},y_{2j},\cdots \;,y_{sj}\right)}^{T},\;j=1,2,\cdots \;,n, \end{array} \end{array}$$

Of which,

The CCR model was as follows:


$$\begin{array}{l} (D_{C^{2}R})\{\begin{array}{l} \min\theta =V_{D},\\ s.t.\;\sum_{j=1}^{n}x_{j}{\text{λ}}_{j}+s^{-}=\theta x_{0},\\ \;\sum_{j=1}^{n}y_{j}{\text{λ}}_{j}-s^{-}=y_{0},\\ \;s^{-}\geq 0,s^{+}\geq 0,{\text{λ}}_{j}\geq 0,j=1,2,\cdots ,n. \end{array} \end{array}$$

$\text{λ}={\left({\text{λ}}_{1},{\text{λ}}_{2},\cdots \;,{\text{λ}}_{n}\right)}^{T}$ was the constant vector of *n* × 1, which was the position weight that calculated the efficiency of the DMUs; *s*^−^ was the slack variable; *s*^+^ was the remaining variable. The optimal solution of the model represented the efficiency value with θ^*^ of (0, 1].

The BCC model added convexity constraints based on the CCR model, which represented assumption of variable returns to scale, and its linear programming was as follows:


$$\begin{array}{l} (D_{BC^{2}})\{\begin{array}{l} \min\theta =V_{D},\\ s.t.\;\sum_{j=1}^{n}x_{j}{\text{λ}}_{j}+s^{-}=\theta x_{0},\\ \;\sum_{j=1}^{n}y_{j}{\text{λ}}_{j}-s^{-}=y_{0},\\ \;\sum_{j=1}^{n}{\text{λ}}_{j}=1,\\ \;s^{-}\geq 0,s^{+}\geq 0,{\text{λ}}_{j}\geq 0,j=1,2,\cdots ,n. \end{array} \end{array}$$

The optimal solutions to the linear programming problems are described below:

(1) If θ^0^ = 1 and *s*^−^ = 0, *s*^+^ = 0, the decision unit *DMU*_*j*_0__ was efficient for DEA. In this case, the production activities of the decision-making unit were scale-efficient and technically efficient.
(2) If θ^0^ = 1, and *s*^−^+*s*^+^>0, the decision unit *DMU*_*j*_0__ was slightly efficient for DEA. In this case, the production activities of the decision-making unit were not scale-efficient and technically efficient at the same time.
(3) If θ^0^ <1, the decision unit *j*_0_ was not efficient for DEA. In this case, the production activities of the decision-making unit were not scale-efficient and technically efficient.

The scale return status of each DMU in the variable returns to scale (BBC) model could be judged by the constant returns to scale (CCR) model. The evaluated DMU was as follows:

(1) ∑λ^*^ <1, indicating that the DMU was in the IRS;
(2) ∑λ^*^ = 1, indicating that the DMU was in the CRS;
(3) ∑λ^*^>1, indicating that the DMU was in the DRS.

Since the BCC model always envelopes the data more rigorously than the CCR model (input-oriented), inefficient hospitals had shorter distances to the boundary in the BCC than the CCR model (43). Herein, the CCR model was used to measure the comprehensive TE, and the BCC model was used to measure PTE and scale efficiency (SE), wherein SE reflected the inefficient parts resulting from the given scale of operation, measured by the ratio of CRS TE scores to VRS TE scores (Equation 5).


(5) $$\begin{array}{l} CCRscore=BCCscore\;\times \;Scale\;efficiency\;\\ \;TE=PTE\;\times \;SE \end{array}$$

DEA model has two types: input-oriented and output-oriented. The output-oriented DEA model aimed to maximize the output using a specific amount of input, while the input-oriented DEA model focused on minimizing the input while ensuring a certain amount of output. Nonetheless, the input-oriented DEA model was suitable for this study because health system managers were more inclined to adjust the resources to achieve optimal hospital performance than to improve the delivery of care under existing medical conditions. In the present study, the DEA-CCR model was used to evaluate the comprehensive technical efficiency of public hospitals of different levels (secondary and tertiary) and different types (comprehensive and specialized) in Fujian Province, and the comprehensive efficiency was resolved using the DEA-BCC model to obtain PTE.

One of the limitations of the DEA model was that the achieved efficiency value was relative to a correlation between sequences. Therefore, the estimated efficiency score of one decision-making unit was not independent of other decision-making units. To address this limitation, Tobit regression was used in the second stage to explore the factors influencing the operation efficiency of the public hospital. The basic model was as follows:


$$\begin{array}{l} \begin{array}{l} y_{i}{}^{*}=a+\beta_{i}x_{i}+\varepsilon_{i},\varepsilon_{i}\sim N(0,\sigma^{2}),i=1,2,3,\cdots ,n\\ y_{i}=\{\begin{array}{l} y_{i}{}^{*}\text{, }0<y_{i}{}^{*}<1\\ 0\text{ , }y_{i}{}^{*}\leq 0\\ 1\text{ , }y_{i}{}^{*}\geq 1 \end{array} \end{array} \end{array}$$

of which, *x*_*i*_ was the explanatory variable; *y*_*i*_ was the predicted variable; β_*i*_ was the unknown parameter; σ^2^ was the estimated parameter.

In the production process, the role of exogenous or environmental factors must also be considered. These factors are not controlled by the organization providing medical care, but may affect the production process of medical care (45).

## 3. Results

### 3.1. Selection of input and output indicators

In order to ensure the credibility and objectivity of data indicators, the combination of qualitative and quantitative methods was adopted for indicator selection.

The combination of the gray rational degree and CV (Table 2) method excluded the indicators *X*4 (medical care ratio), *Y*3 (bed utilization rate), *Y*4 (number of bed turnovers), *Y*5 (average hospital stay), and *Y*6 (average cost of outpatient (emergency) visits) according to the principle that the mean gray rational degree was >0.75 and the coefficient of variation was >0.6.

**Table 2:** Average gray rational degree and coefficient of variation of each indicator.

**Indicator**	**ε_*i*_**	**CV**	**Indicator**	**ε_*i*_**	**CV**
*X*1	0.8536	0.9005	*Y*3	0.7262	0.3530
*X*2	0.8592	0.8710	*Y*4	0.7603	0.4269
*X*3	0.8592	0.8993	*Y*5	0.7207	0.3240
*X*4	0.6714	0.3240	*Y*6	0.7874	0.4646
*X*5	0.7633	0.8325	*Y*7	0.7874	0.6051
*X*6	0.7633	0.8112	*Y*8	0.8229	1.2273
*X*7	0.9284	1.0979	*Y*9	0.7726	1.4291
*X*8	0.9020	1.0946	*Y*10	0.8203	1.1265
*X*9	0.9295	1.2154	*Y*11	0.7684	1.0278
*X*10	0.8882	1.4908	*Y*12	0.8880	1.1031
*Y*1	0.7834	0.9236	*Y*13	0.8825	1.1328
*Y*2	0.7834	1.0083	*Y*14	0.7691	1.1223

The gray clustering showed that *X*1, *X*5, and *X*6 were clustered into one group, *X*7 and *X*9 were clustered into one type, *Y*2, *Y*12, and *Y*13 could be clustered together, and *Y*3 and *Y*14 were clustered in a group. Subsequently, only one indicator of one type was selected. Dyson et al. (43) demonstrated that absolute data should not be mixed with relative data, otherwise the results may be severely distorted. Thus, *X*4 and *Y*3, as the only relative data of the input indicators and output indicators, respectively, should be eliminated. The selected input indicators and output indicators were finally determined (Table 3).

**Table 3:** Input and output indicators.

**Variables**	**Definition of variable**
**Input indicators**	
Number of physicians	People who have licensed physicians with the “Physician Practice Certificate” at the end of the year and are engaged in medical and preventive health care work
Number of nurses	People who have the registered nurse certificate at the end of the year and are engaged in nursing work
Number of hospital beds	The number of beds approved by the health administration in the current year
Personnel expenditure	Includes the basic salary, performance pay, allowance, social, and insurance contributions of personnel but does not include the subsidy expenditure for individual families
Public administration expenditure	Refers to the expenditure of administrative units for the maintenance of equipment and facilities for the completion of work tasks and expenditures directly used for official activities
**Output indicators**	
Outpatient visits	The number of people who are not hospitalized, which is the sum of the number of outpatient and emergency patients
Average cost of hospitalization	Medical expenses per discharge, i.e., medical inpatient income/number of discharged
Drug revenue	Revenue from drug sales charged by hospitals to patients
Total income	Non-reimbursable funds obtained by the hospital according to the law for business purposes and other activities

**Figure 1:**
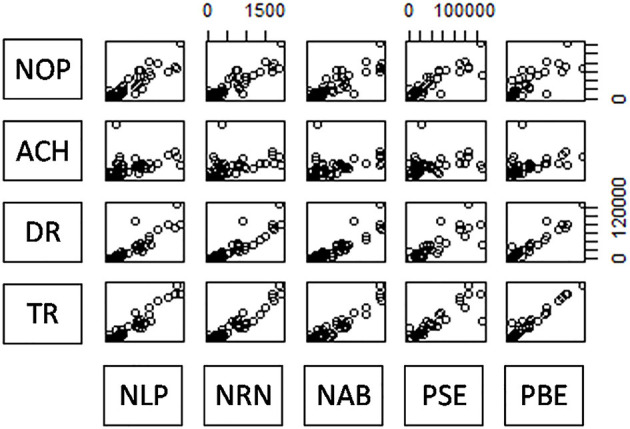
Scatterplot matrix of input and output indicators. NOP-PAE represents the number of physicians, nurses, beds, personnel expenditure, and public management expenditure. OV-TI represents the number of outpatient visits, the average cost of hospitalization, drug revenue, and total income.

The scatterplot matrix showed a high correlation between input indicators and output indicators (Figure 1), which met the data homobosity requirements of the DEA model. Among these, row variables represent input indicators and column variables represent output indicators.

### 3.2. Selection of Tobit regression influencing factors

In order to study the factors affecting the operational efficiency of public hospitals in Fujian Province, we considered institutional factors (i.e., factors that can be controlled through hospital management) and background factors (i.e., factors beyond the control of hospital management) and identified six regression variables to assess their impact on hospital performance, according to Orsini et al. (35) (Table 4).

**Table 4:** Variables of Tobit regression.

**Variables**	**Definition of variable**
**Institutional factors**	
Bed occupancy rate (52)	The size of the hospital beds relative to the number of inpatients. The bed occupancy rate = hospital stay (day)/(365 × number of beds) × 100
Average length of stay (35)	Total hospital stay (days)/number of inpatients
Hospital bed size (36)	This is a dummy variable with 1 for >1,000 beds, 0 for ≤ 1,000 beds
Proportion of beds (52)	Number of public hospital beds/total number of beds in all public hospitals × 100
**Environmental factors**	
GDP per capita (29)	Total GDP/Population
Proportion of government subsidies in hospital income (4)	Government financial allocation/total hospital revenue × 100

In order to avoid possible heteroscedasticity in the data, the bed occupancy rate, the average length of stay, the proportion of beds, the GDP per capita, and the proportion of government subsidies in hospital income were logarithmized.

Based on definition, the DEA score was between 0 and 1, and some data focused on the boundary value of 1. Thus, DMU with a value of 1 should be reviewed (46). According to the study by Zere (47), the DEA efficiency score was converted into an inefficiency score by the following formula, assuming 0 as the review point:


$$\begin{array}{l} inefficiency\;score=\frac{1}{DEA\;score}-1 \end{array}$$

Therefore, the efficient decision unit had a score of 0, and the inefficient decision unit had a score >0.

**Figure 2:**
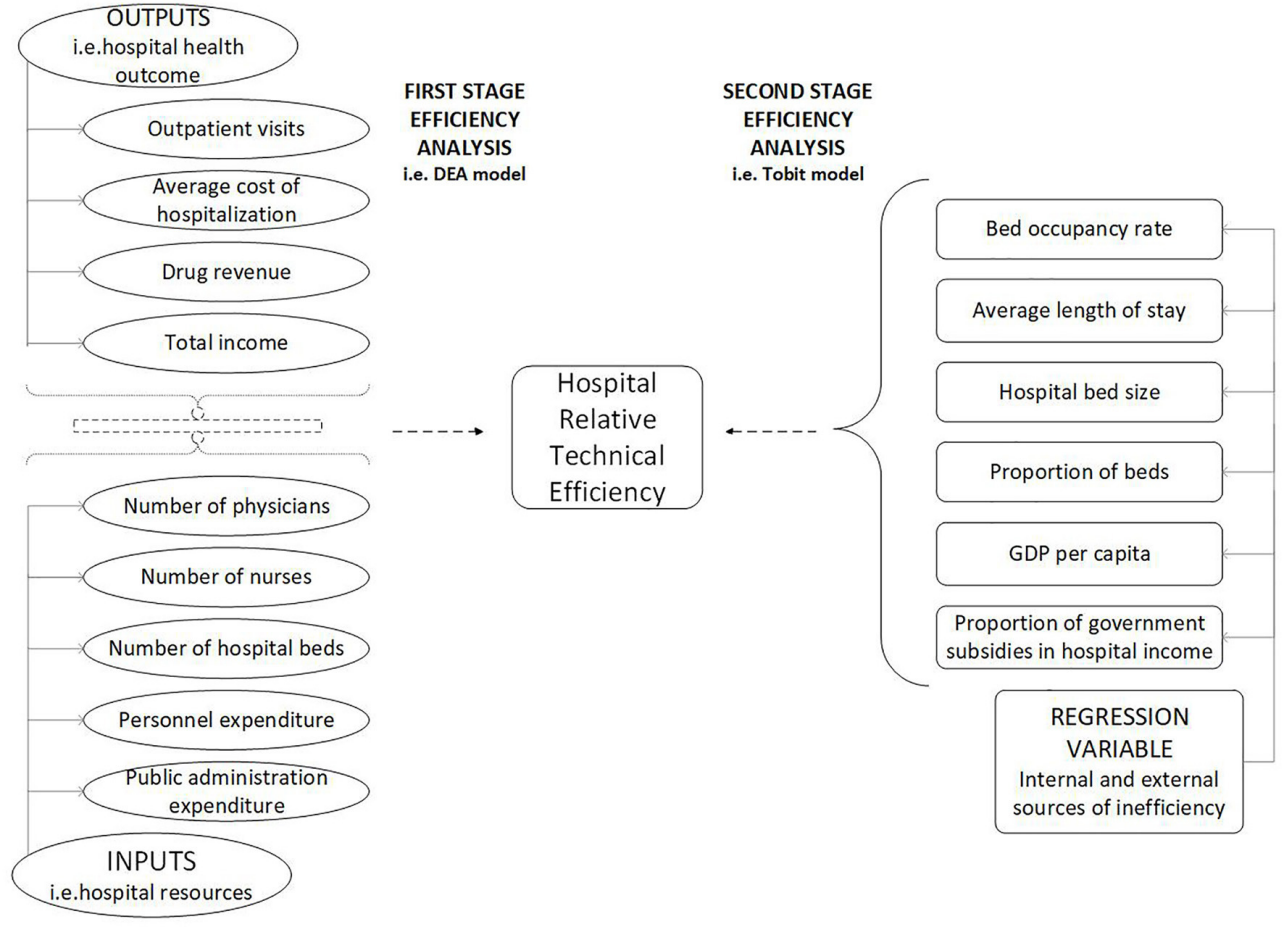
Conceptual production structure diagram of hospitals.

The Tobit regression model was represented as follows:


$$\begin{array}{l} Inefficiency_{i}=\beta_{0}+\beta_{1}BOR_{i}+\beta_{2}ALOS_{i}+\beta_{3}SIZE_{i}+\;\\ \;\beta_{4}POB_{i}+\beta_{5}GDPPC_{i}+\beta_{6}PGSH_{i}+\varepsilon_{i} \end{array}$$

wherein, Inefficiency indicated the inefficiency score; BOR indicated bed occupancy rate; ALOS indicated average length of stay; SIZE indicated hospital bed size;POB indicated proportion of beds, which suggested the number of public hospital beds as a percentage of the total number of beds in all public hospitals; GDPPC indicated region GDP per capita;PGSH indicated the proportion of government subsidies in hospital revenue; ε was the random error. The flow chart of two-stage data envelopment analysis is shown in Figure 2.

### 3.3. Efficiency evaluation of public hospitals

Among the 49 sample hospitals, 36 were grade III and 13 were grade II hospitals; 37 were general and 12 were specialized hospitals. The DEA-BCC model was used to calculate the scores of comprehensive TE, PTE, and SE of 49 public hospitals in Fujian Province; and the mean values were 0.802, 0.887, and 0.903, respectively (Table 5). The distribution of efficiency values is shown in Table 6.

**Table 5:** Efficiency scores and scale reward status of 49 public hospitals.

**Hospital name**	**CRS technical efficiency**	**VRS technical efficiency**	**Scale efficiency**	**Returns to scale**
H1	0.833	0.939	0.887	DRS
H2	1.000	1.000	1.000	–
H3	0.884	1.000	0.884	DRS
H4	1.000	1.000	1.000	–
H5	0.842	0.843	0.999	–
H6	1.000	1.000	1.000	–
H7	1.000	1.000	1.000	–
H8	0.825	1.000	0.825	DRS
H9	0.724	0.739	0.979	DRS
H10	0.798	0.902	0.885	DRS
H11	0.939	1.000	0.939	DRS
H12	0.741	0.774	0.957	DRS
H13	0.901	0.964	0.934	DRS
H14	1.000	1.000	1.000	–
H15	0.836	1.000	0.836	DRS
H16	0.77	1.000	0.77	DRS
H17	0.885	1.000	0.885	DRS
H18	0.607	0.638	0.952	DRS
H19	0.606	0.724	0.836	DRS
H20	1.000	1.000	1.000	–
H21	1.000	1.000	1.000	–
H22	0.902	1.000	0.902	DRS
H23	0.589	0.662	0.889	DRS
H24	0.453	0.607	0.746	DRS
H25	0.690	0.979	0.705	DRS
H26	0.568	0.783	0.725	DRS
H27	0.785	1.000	0.785	DRS
H28	0.788	0.858	0.918	DRS
H29	0.796	0.852	0.935	DRS
H30	0.742	1.000	0.742	DRS
H31	0.596	0.646	0.923	IRS
H32	0.736	0.771	0.955	IRS
H33	0.750	0.874	0.858	DRS
H34	0.658	0.725	0.907	DRS
H35	1.000	1.000	1.000	–
H36	1.000	1.000	1.000	–
H37	0.736	0.793	0.929	DRS
H38	0.928	1.000	0.928	DRS
H39	0.814	0.817	0.996	DRS
H40	1.000	1.000	1.000	–
H41	0.772	1.000	0.772	DRS
H42	0.576	0.671	0.858	DRS
H43	0.607	0.807	0.753	DRS
H44	0.747	0.929	0.804	DRS
H45	0.774	0.862	0.898	DRS
H46	0.832	0.974	0.855	DRS
H47	1.000	1.000	1.000	–
H48	0.809	0.898	0.902	DRS
H49	0.435	0.447	0.972	IRS
Mean	0.802	0.887	0.903	

DRS indicated decreasing returns to scale, IRS indicated increasing returns to scale, and – indicates constant returns to scale.

**Table 6:** Efficiency distribution of public hospitals in Fujian Province.

**Efficiency value**	**Comprehensive TE**		**PTE**		**SE**	
***N***	**%**	***N***	**%**	***N***	**%**
<0.6	6 (2)	12.24	1 (1)	2.04	0 (0)	0
0.6–0.7	5 (1)	10.20	5 (1)	10.20	0 (0)	0
0.7–0.8	14 (4)	28.57	7 (1)	14.29	8 (2)	16.33
0.8–0.9	9 (1)	18.37	8 (3)	16.33	13 (1)	26.53
0.9–1	4 (1)	8.16	6 (1)	12.24	17 (6)	34.69
1	11 (4)	22.45	22 (6)	44.90	11 (4)	22.45
Total	49 (13)	100	49 (13)	100	49 (13)	100

Number in parentheses indicates the number of grade II hospitals.

Among the 49 public hospitals, 11 had a PTE of 1 and SE of 1; hence, the overall hospital effective rate was 22.45%, i.e., 22.45% of the hospitals were both technically effective and scale-effective, indicating that they were at the production frontier of all public hospitals in Fujian Province. Among these 4 were grade II hospitals, accounting for 30.77% of the total number of 13 grade II hospitals, and 7 were grade III hospitals, accounting for 19.44% of the total number of 36 grade III hospitals. There were 4/12 (33%) specialized hospitals and 7/37 (18.92%) general hospitals. Together, the overall efficiency of secondary public hospitals in Fujian Province was higher than that of tertiary public hospitals, while the overall efficiency of specialized hospitals was higher than that of general hospitals.

**Figure 3:**
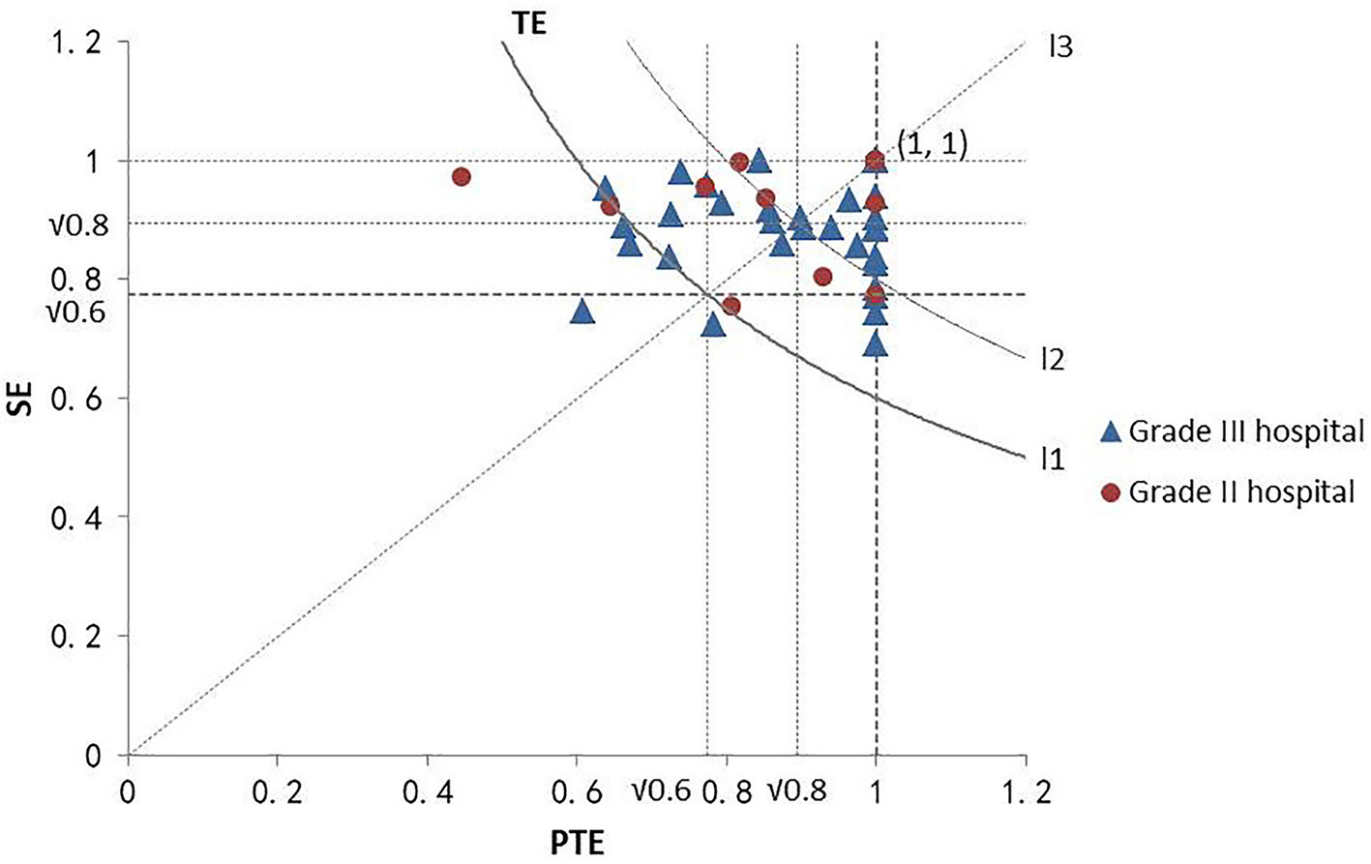
DEA efficiency distribution of hospitals at different grades. The point at the bottom left of curve *L*_1_ represents the comprehensive TE of the hospital at <0.6. The point on curve *L*_1_ represents the comprehensive TE of the hospital as 0.6. The point between curves *L*_1_ and *L*_2_ represents the comprehensive TE of the hospital between 0.6 and 0.8, and the point on the upper right of curve *L*_2_ represents the comprehensive TE of the hospital >0.8. The point above line *L*_3_ represents SE greater than PTE. The point on line *L*_3_ represents the SE was equal to PTE, and the point below line *L*_3_ represents SE lower than PTE.

In order to analyze the efficiency distribution of public hospitals in Fujian Province, the efficiency distribution of each hospital could be located in the cartesian coordinate system by taking the DEA PTE as the horizontal axis and the SE as the vertical axis (Figure 3). Since DEA comprehensive TE was the product of PTE and SE, the comprehensive TE from the bottom left to the upper right was consistently improved in the coordinate system in the figure. Also, the PTE and SE of the hospital with the coordinates (1,1) were 1, which marked it as an effective hospital. Hospitals with other points were classified as inefficient. Mehrtak et al. (48) divided the comprehensive TE value into three levels: inefficient, slightly inefficient, and efficient. Figure 3 shows that among the grade II hospitals, the comprehensive TE of two hospitals was <0.6, the comprehensive TE of five hospitals was between 0.6 and 0.8, and the comprehensive TE of six hospitals was >0.8, *viz*, 15.38% of grade II hospitals were inefficient, 38.46% of hospitals were slightly efficient, and 46.15% of hospitals were efficient. Among the grade III hospitals, 11.11% were inefficient, 38.89% were slightly inefficient, and 50% were efficient. Moreover, the SE of grade II hospitals was equivalent to PTE, while the PTE of grade III hospitals was significantly higher than the SE. Typically, the comprehensive TE of grade II public hospitals in Fujian Province was higher than that of grade III public hospitals.

Heterogeneity was detected in operational efficiency due to varied environments of different levels and types of hospitals. Previous studies did not consider the differences among hospitals with different levels and types, reducing the applicability of the results. The present study analyzed grade II and grade III hospitals, general and specialized hospitals, respectively (Table 7). The average PTE and SE of general hospitals were higher than those of specialized hospitals, indicating that the scale and technical management of grade II general hospitals were better than that of grade II specialized hospitals. Among grade III hospitals, the average PTE of general hospitals was higher than that of specialized hospitals, while the average SE was lower than that of specialized hospitals, indicating that the scale management of grade III specialized hospitals was better than that of grade III general hospitals, but the technical level of grade III specialized hospitals was not as good as that of grade III general hospitals. Among general hospitals, the average PTE of grade III hospitals was higher than that of grade II hospitals, but the average SE of grade III hospitals was lower than that of grade II hospitals, indicating that the excessive scale of grade III general hospitals affected the improvement of their efficiency. Among the specialized hospitals, the average PTE and the average SE of grade III hospitals were higher than those of specialized hospitals, indicating that the scale management and technical level of grade III specialized hospitals were better than those of grade II specialized hospitals.

**Table 7:** Average efficiency of public hospitals with different levels and types.

**Efficiency value**	**Comprehensive TE**		**PTE**		**SE**	
**Grade II hospital**	**Grade III hospital**	**Grade II hospital**	**Grade III hospital**	**Grade II hospital**	**Grade III hospital**
General-hospitals	0.8249 (0.1463)	0.7981 (0.1255)	0.8893 (0.1216)	0.9047 (0.1125)	0.9262 (0.0871)	0.8826 (0.0843)
Specialized-hospitals	0.7273 (0.2311)	0.8103 (0.1858)	0.7920 (0.2457)	0.8647 (0.1574)	0.9253 (0.0866)	0.9286 (0.0791)
Total	0.8024 (0.1746)	0.8012 (0.1431)	0.8668 (0.1643)	0.8947 (0.1264)	0.9260 (0.0870)	0.8941 (0.0854)

Number in parentheses indicates standard deviations.

Further analysis used the same output model and assumed that all hospitals had the same output to measure the gap between the input index value of ineffective hospitals and the target value of indicators under the condition of efficiency; consequently, the redundancy between the actual value of input resources of the ineffective hospital and the target value was obtained (Table 8). The negative sign indicated that the decision-making unit needs to reduce the input to achieve the effective state. For grade II hospitals, if the hospital operation achieved relative effectiveness, the average number of physicians needed to be reduced by 37.02%, the number of nurses needed to be reduced by 51.24%, and the number of hospital beds needed to be reduced by 37.27%. Similarly, personnel expenditure and public administration expenditure should be reduced by 34.35 and 24.73%, respectively, for the best use of resources. Similarly, for grade III hospitals, the number of physicians needs to be reduced by 25.45%, the number of nurses needs to be reduced by 30.67%, the number of beds needs to be reduced by 29.32%, and the personnel expenditure and public administration expenditure should be reduced by 20.51 and 19.66%, respectively. The comparison found that among the ineffective hospitals, grade III public hospitals had more investment redundancy than grade II public hospitals in terms of human, material, and financial resources.

**Table 8:** Relaxation assessment of average input of ineffective hospitals.

**DMU**	**NOP**	**NON**	**NOB**	**PE**	**PAE**
H1	−6.09%	−6.11%	−12.35%	−6.09%	−6.09%
H5	−15.73%	−30.87%	−43.19%	−15.73%	−15.73%
H9	−51.21%	−26.02%	−27.11%	−26.06%	−26.06%
H10	−18.26%	−17.38%	−14.32%	−9.80%	−9.80%
H12	−62.20%	−22.73%	−31.63%	−22.61%	−22.61%
H13	−25.42%	−19.63%	−3.57%	−3.57%	−3.57%
H18	−36.16%	−36.12%	−37.25%	−36.19%	−36.19%
H19	−27.59%	−28.74%	−37.21%	−27.59%	−27.59%
H23	−33.95%	−33.72%	−37.24%	−33.80%	−33.80%
H24	−39.29%	−61.42%	−73.34%	−39.29%	−39.29%
H25	−2.13%	−28.63%	−28.83%	−2.14%	−10.96%
H26	−29.25%	−33.62%	−51.40%	−21.72%	−21.72%
H28	−23.32%	−23.78%	−14.16%	−14.16%	−14.16%
H29	−28.88%	−29.88%	−26.39%	−14.80%	−14.80%
H31	−35.61%	−50.90%	−35.42%	63.55%	−35.42%
H32	−55.95%	−70.12%	−22.95%	−27.66%	−22.95%
H33	−12.90%	−38.22%	−20.21%	−12.55%	−12.55%
H34	−32.44%	−37.96%	−27.47%	−58.62%	−27.47%
H37	−24.47%	−20.78%	−22.05%	−20.75%	−20.75%
H39	−18.80%	−46.98%	−65.18%	−18.27%	−18.27%
H42	−32.86%	−51.63%	−44.71%	−32.91%	−32.91%
H43	−41.00%	−48.52%	−34.29%	−19.34%	−19.34%
H44	−23.56%	−30.45%	−7.06%	−39.05%	−7.06%
H45	−13.79%	−23.69%	−31.58%	−13.82%	−13.82%
H46	−11.71%	−35.65%	−2.60%	−2.60%	−7.97%
H48	−10.31%	−36.76%	−26.22%	−10.21%	−10.21%
H49	−55.37%	−81.85%	−69.58%	−57.77%	−55.31%
Grade II hospitals	−37.02%	−51.24%	−37.27%	−34.35%	−24.73%
Grade III hospitals	−25.45%	−30.67%	−29.32%	−20.51%	−19.66%
Average	−28.45%	−36.01%	−31.38%	−24.10%	−20.98%

NOP represents the number of physicians, NON represents the number of nurses, NOB represents the number of beds, PE represents personnel expenditure, and PAE represents public administration expenditure.

In terms of scale remuneration, 69.39% of public hospitals have decreased scale compensation, 24.49% of public hospitals have constant scale compensation, and 6.12% of public hospitals have increased scale compensation. From the perspective of hospital grade, the proportion of hospitals with decreasing scale remuneration in grade III hospitals was much higher than that of grade II hospitals, indicating that the scale of grade III hospitals was too large, which was not conducive to the improvement of their comprehensive efficiency. From the perspective of the type, the proportion of hospitals with increasing scale remuneration of specialized hospitals was higher than that of general hospitals, indicating that the appropriate increase in the operation scale of specialized hospitals was conducive to the improvement of comprehensive efficiency (Table 9).

**Table 9:** Scale remuneration of public hospitals in Fujian Province.

**Scale remuneration**	**Overall samples**		**Grade II hospitals**		**Grade III hospitals**		**General hospitals**		**Specialized hospitals**	
***n***	**%**	***n***	**%**	***n***	**%**	***n***	**%**	***n***	**%**
Constant	12	24.49	4	30.77	8	22.22	8	21.62	4	33.33
Increasing	3	6.12	3	23.08	0	0	2	5.41	1	8.33
Decreasing	34	69.39	6	46.15	28	77.78	27	72.97	7	58.33
Total	49	100	13	100	36	100	37	100	12	100

### 3.4. Analysis of factors influencing the efficiency of public hospitals

In this study, the comprehensive TE and PTE of public hospitals in Fujian Province were considered as the dependent variables, and the institutional and background factors selected above were taken as independent variables. A Tobit regression model was established to analyze the influencing factors of CCR and BCC efficiencies of grade II and III public hospitals, respectively. The results showed the Tobit regression coefficients and testing results (Table 10).

**Table 10:** Tobit regression of DEA-CCR and DEA-BCC models.

**Tobit regression**	**CCR model (TE)**		**BCC model (PTE)**	
**Model 1 (grade III hospitals)**	**Model 2 (grade II hospitals)**	**Model 3 (grade III hospitals)**	**Model 4 (grade II hospitals)**
BOR	−1.385^***^ (0.436)	−0.529 (0.575)	−1.146^**^ (0.457)	−0.033 (0.842)
ALOS	0.716^**^ (0.281)	0.807^*^ (90.365)	0.970^***^ (0.304)	1.126^*^ (0.497)
SIZE	−0.281^**^ (0.119)	–	−0.205^*^ (0.118)	–
POB	0.771^***^ (0.229)	0.931^**^ (0.363)	0.430^*^ (0.223)	0.468 (0.478)
GDPPC	−0.546^**^ (0.237)	−0.831^**^ (0.540)	−0.661^**^ (0.267)	−0.572 (0.734)
PGSH	0.251^**^ (0.121)	0.112 (0.130)	0.160 (0.118)	0.176 (0.179)
Constant	2.558 (0.786)	1.449 (0.986)	1.919 (0.823)	−0.368 (1.399)
Observation	36	13	36	13
Sigma	0.014	0.023	0.015	0.049
Pseudo *R*^2^	0.682	0.895	0.608	0.587
Log likelihood	−4.930	−1.038	−6.396	−4.265

^*^p <0.1, ^**^p <0.05, ^***^p <0.01. Standard errors are shown in parentheses. BOR represents bed occupancy rate; ALOS represents average length of stay; SIZE represents hospital bed size; POB represents proportion of beds; GDPPC represents region GDP per capita; PGSH represents the proportion of government subsidies in hospital revenue.

The regression results of grade III hospitals showed that in the CCR model, the effects of bed occupancy rate and proportion of beds on the comprehensive TE of grade III hospitals were statistically significant at the level of 1%, and the increased bed occupancy rate had a negative effect on the inefficiency of tertiary hospitals and the effect of proportion of beds on the inefficiency of grade III hospitals was positive. Importantly, grade III public hospitals with high bed occupancy rate had high comprehensive TE, while higher proportion of beds could hinder the further improvement of TE. In addition, the effects of average length of stay, hospital bed size, GDP per capita, and proportion of government subsidies in hospital income had statistically significant effects on the comprehensive TE of grade III hospitals at the level of 5%. With an increase in the average hospital stay and the proportion of government subsidies in hospital income, the comprehensive TE of grade III hospitals decreased. With the increasing size of hospital beds and regional GDP per capita, the comprehensive TE of grade III hospitals increased. In the BCC model, only the effect of average length of stay on the PTE of grade III hospitals was significant at the level of 1%. With the increase in bed occupancy rate, hospital bed size and GDP per capita, the PTE of tertiary hospitals increased. With the increase in average hospital stay, the proportion of beds and the proportion of government subsidies in hospital income, the PTE of grade III hospitals decreased.

**Figure 4:**
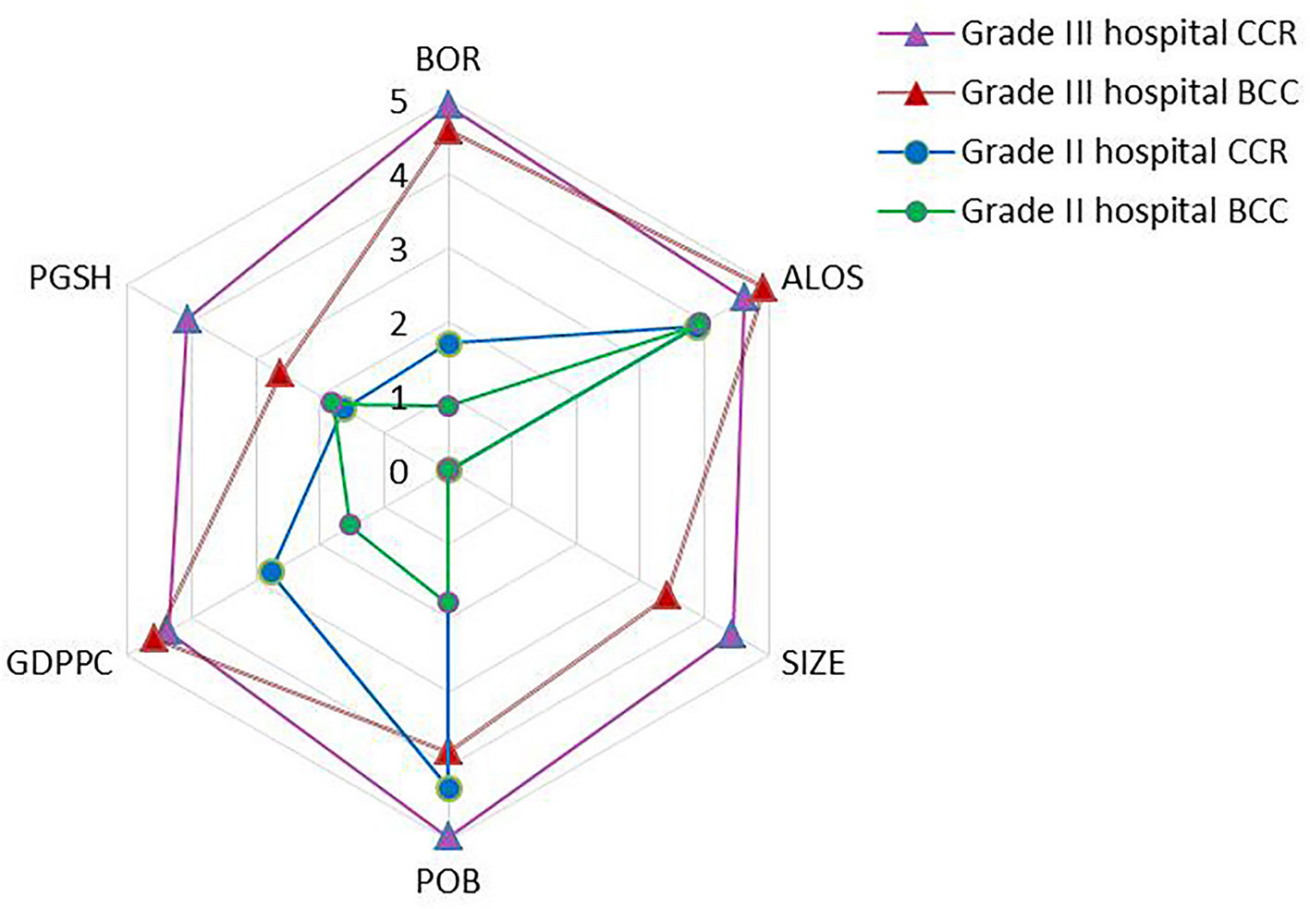
Comparison of the significance of environmental factors.

Compared to grade III public hospitals, the bed occupancy rate, hospital bed size, and the proportion of government subsidies in hospital income did not have a significant impact on the efficiency of grade II hospitals. The results showed that the influencing factors of different levels of comprehensive TE varied. The significance level in Figure 4. was the result of reverse processing of the critical value of the model regression coefficient test. The higher the level value, the more significant the influence of the factor on the comprehensive TE.

In the CCR model, the comprehensive TE of grade II hospitals decreased with the increase in average length of stay and the proportion of beds. With the increase in GDP per capita, the comprehensive TE of grade II hospitals increased, while in the BCC model, the PTE of grade II hospitals decreased with the increase in the average length of stay. The regression results of CCR and BCC models did not show any significant effects of hospital bed size on the efficiency value of grade II public hospitals. Because the values of hospital bed size variables of all grade II hospitals were 0, the number of beds in all grade II sample hospitals was <1,000, and the regression results of the influencing factors on hospital bed size in the four models only provided reference values for grade III hospitals.

## 4. Discussion

In order to assess hospital efficiency, most scholars only use a qualitative or quantitative method to select indicators. Due to the complex environment of the medical system, there are many optional indicators. In order to ensure the representativeness of indicators, we regard the medical system as a gray system. With the help of gray correlation and gray clustering analysis methods, we combine qualitative and quantitative methods to select indicators.

Recently, most of the studies on public hospitals in China only consider tertiary hospitals or primary medical institutions, and few of them put secondary hospitals or above together. Public hospitals in China are divided into different levels according to the size of beds. Different levels of hospitals have the same resource conditions, but face different development opportunities. Therefore, it is necessary to compare the differences in the amount and efficiency between hospitals of different levels, It can better promote the improvement and perfection of public hospitals. In order to analyze the operational efficiency of public hospitals in Fujian Province, we evaluated the efficiency of different types and levels of public hospitals and the institutional and environmental factors related to efficiency, such as bed occupancy rate, average hospital stay, hospital bed size, proportion of beds, regional per capita GDP, and government subsidies to hospital revenue. Most of the available literature has only focused on the comparison of public and private hospitals or evaluated the efficiency of public hospitals in different administrative units. Thus, the present study evaluated the operational efficiency of different types and levels of public hospitals rather than the operational efficiency of hospitals in different administrative regions.

The results showed that among the 49 sample public hospitals in Fujian Province in 2020, 11 were located at the production frontier. The proportion of effective hospitals is 22.45%, which was low. Although only 22.45% of hospitals had the scale efficiency of 1, the proportion of hospitals with pure technical efficiency of 1 reached 46.94%, indicating that 24.49% of hospitals hindered the improvement of comprehensive efficiency value due to low-scale efficiency value, suggesting that public hospitals in the Fujian Province had the problem of hindered operational efficiency due to oversize. This result was consistent with that reported by Kirigia and Asbu (49), showing that most public hospitals need to reduce their size to improve efficiency.

Among the 13 high-efficiency hospitals included in this study (excluding the hospitals with TE of 1), 12 (92.31%) exhibited decreasing returns to scale, suggesting that although they were the most efficient, there was a large amount of over-resourced production system in the hospitals. Similarly, 18/19 slightly inefficient hospitals (94.74%) had decreasing returns to scale. Among the 6 inefficient hospitals, 4 (66.67%) had decreasing returns to scale, showing that although the operation efficiency of the inefficient hospitals was not high, the production of some hospitals was carried out under increasing returns to scale. The grading study showed that the SE of grade II hospitals was equivalent to the PTE, and the PTE of grade III hospitals was significantly greater than the SE, indicating that the problem of the excessive scale of public hospitals in Fujian Province was mainly caused by grade III hospitals.

Tobit regression analysis found that the effect of bed occupancy rate on the comprehensive TE of grade III hospitals was statistically significant at the level of 1%, indicating that the higher the bed occupancy rate of the hospital, the higher the efficiency value of the hospital, which was consistent with the finding by Orsini et al. (35). Similarly to the results of Dimas et al. (50), the present study showed that the average stay was one of the main reasons hindering the improvement of comprehensive TE in hospitals. In addition, this study found that some policy variables, such as the proportion of government subsidies in hospital income and the GDP per capita of development level indicators, have positive effects on the technical efficiency of hospitals, while the proportion of beds restrained the improvement of technical efficiency, i.e., public hospitals in Fujian Province had the issue of excessive beds and hindered operational efficiency.

Our research has several advantages: First, this is the first paper to study the influencing factors of public hospitals at different levels in China. This research provides empirical evidence for national public hospital evaluation research and practical suggestions for public hospital reform. In addition, we combine qualitative and quantitative methods when selecting indicators. This scientific method avoids the defects of single method. Third, our research compares the efficiency of public hospitals at different levels, and points out the problems in the current management of public hospitals in China. It also explores the institutional and environmental factors that affect the low efficiency of public hospitals.

Our research has several limitations: First, we unable to explain the long-term impact of institutional and environmental factors on hospital inefficiency due to a lack of available panel data. However, our research results are still useful to assess the short-term impact of hospital inefficiency. In the future, research on data sets with different time dimensions will yield more interesting facts. Secondly, since the complete data of the study population cannot be obtained, our study is based on sample data. However, since our sample includes hospitals of all levels and types in all cities, it has the same structure as the research population and is representative of the population. Third, we only have data on hospitals in one provincial, which limits the generalization of our results. Although this limitation is very common in studies, we are lucky to include the hospitals in our study province. In addition, the medical development level of our study province ranks in the middle of the country and is representative of the whole China in terms of the average level of economic and social development. Therefore, our findings are still applicable to public hospitals in all provinces of China.

## 5. Conclusion

This study analyzed the operational efficiency of 49 hospitals and discussed the influencing factors of hospitals at different levels. The results showed that the overall operational efficiency of public hospitals in Fujian Province was low, and most hospitals had redundant resources. Tobit regression analysis showed that government subsidies and regional economic development affected the operational efficiency of hospitals. As Clemens et al. (51) studied EU hospitals, this study suggests that managers solve health system problems through hospital structure reform. To alleviate current problems and improve the operational efficiency of hospitals, several strategies are suggested for hospital managers and relevant government agencies.

We put forward several suggestions to improve the utilization efficiency of medical resources. First of all, the managers of inefficient hospitals should follow the best performing hospitals at the same level when possible, and find the appropriate proportion of investment according to their specific conditions. In addition, relevant departments should reasonably allocate the resources of each hospital, appropriately develop secondary public hospitals, and control the further expansion of tertiary public hospitals to maximize the use of resources. Finally, hospital managers should formulate talent introduction plans, build a reasonable talent echelon, and increase the introduction of nursing staff and high-level personnel.

The management department should vigorously develop specialized hospitals to make them play a full role in the medical system. In addition, on the basis of scientific planning for the size of hospital beds, managers can consider to carry out day hospitals as much as possible, and adequate nursing without overnight care can also increase the occupancy rate of beds.

Certainly, please remember that efficiency is not the ultimate goal of the hospitals, but merely a means through which the primary goal of achieving health output can be supported. In the process of moving toward an efficient hospital, decision-makers must continue to recognize the unique challenges faced by hospitals and bear the burden of inpatient and outpatient care for local residents. In this process, the negative impact of basic services on population health should be minimized.

## Data availability statement

The datasets presented in this study can be found in online repositories. The names of the repository/repositories and accession number(s) can be found below: http://fujian.gov.cn/zwgk/zdlyxxgk/ggws/ylzlxxgs/.

## Author contributions

MS made contributions to study design, developed the methodology, performed the statistical analysis, and wrote the manuscript. GZ developed the methodology and coordinated the analyses. YY, XS, and YX developed the methodology, reviewed, and edited the manuscript. All authors contributed to the article and approved the submitted version.
